# A Comparative Study of Window Size and Channel Arrangement on EEG-Emotion Recognition Using Deep CNN

**DOI:** 10.3390/s21051678

**Published:** 2021-03-01

**Authors:** Panayu Keelawat, Nattapong Thammasan, Masayuki Numao, Boonserm Kijsirikul

**Affiliations:** 1 Department of Computer Science and Engineering, University of California San Diego, La Jolla, CA 92093-0404, USA; pkeelawa@ucsd.edu; 2 Department of Computer Engineering, Chulalongkorn University, Pathum Wan, Bangkok 10330, Thailand; 3 Human Media Interaction, Faculty of Electrical Engineering, Mathematics and Computer Science, University of Twente, 7522 NB Enschede, The Netherlands; n.thammasan@utwente.nl; 4 The Institute of Scientific and Industrial Research, Osaka University, Mihogaoka, Ibaraki, Osaka 567-0047, Japan; numao@sanken.osaka-u.ac.jp

**Keywords:** emotion recognition, EEG, machine learning, CNN, spatiotemporal data, brainwave, neuroscience, window size, electrode order

## Abstract

Emotion recognition based on electroencephalograms has become an active research area. Yet, identifying emotions using only brainwaves is still very challenging, especially the subject-independent task. Numerous studies have tried to propose methods to recognize emotions, including machine learning techniques like convolutional neural network (CNN). Since CNN has shown its potential in generalization to unseen subjects, manipulating CNN hyperparameters like the window size and electrode order might be beneficial. To our knowledge, this is the first work that extensively observed the parameter selection effect on the CNN. The temporal information in distinct window sizes was found to significantly affect the recognition performance, and CNN was found to be more responsive to changing window sizes than the support vector machine. Classifying the arousal achieved the best performance with a window size of ten seconds, obtaining 56.85% accuracy and a Matthews correlation coefficient (MCC) of 0.1369. Valence recognition had the best performance with a window length of eight seconds at 73.34% accuracy and an MCC value of 0.4669. Spatial information from varying the electrode orders had a small effect on the classification. Overall, valence results had a much more superior performance than arousal results, which were, perhaps, influenced by features related to brain activity asymmetry between the left and right hemispheres.

## 1. Introduction

Recognition of emotion from physiological sensors has become a highly active research area, bridging the gap between humans and computers [1]. In this task, electroencephalogram (EEG) is a pragmatic and attractive tool due to its high temporal resolution and cost-effectiveness in measuring the activity of the brain, which is commonly known as the center of the emotion process [2]. EEG has been frequently used to estimate emotional states of humans, which can be represented by the arousal-valence model [3], but the recognition of emotions based solely on brainwaves is still a very challenging task [4] owing to the non-stationary and non-linear characteristics of EEG and inter-subject variability [5], especially when aiming to build a global classifier that is compatible with everyone. Conventional methods that rely on handcraft feature engineering suffer from the subject issue, resulting in limited performance, although a number of sophisticated supervised learning methods have been proposed [6,7,8,9,10,11,12].

Recently, convolutional neural network (CNN), with unmanned feature representation learning, has been applied to EEG-based emotion recognition [13], circumventing the cumbersome feature engineering while improving classification accuracy concurrently [14,15]. It has advantages at capturing adjacent spatial information [16,17], which, in this case, is spatiotemporal material from EEG. Its performance has been found to be superior to other traditional approaches in subject-dependent emotion recognition [18,19] and it demonstrates the potential to generalize the classification to unseen subjects [20]. The network has been applied to numerous problems, including EEG-related tasks, such as motor imagery classification [21], P300 EEG classification [22], sleep stage scoring [23], human activity recognition based on EEG [24], etc. However, CNN-based EEG-emotion recognition is still in its infancy. Apart from trainable CNN parameters, many hyperparameters focusing on spatiotemporal factors of EEG, which are important and can impact the recognition, remain under-explored.

Emotional responses to music can evolve over the course of time, as evidenced by episodes of musical chills [25], and this fact necessitates the continuous annotation of emotion to allow capturing the temporal dynamic of emotion when listening to music [26]. With the aim to train a classifier to recognize time-varying emotion, a sliding window is used, allowing the increment of samples, and the heightened granularity in capturing emotion that evolves over the course of time has helped improve the accuracy of emotion recognition [27]. Nevertheless, the selection of window size is not straightforward. In particular, if a window is too short, the stimulus that induced responses may not have been fully unfolded during the window, while excessively long windows might fail to capture salient emotional response which might be buried by the inclusion of trivial information.

The temporal effect in musical response and annotation is, therefore, of interest in our study. An EEG study investigated event-related potentials of subjects listening to standardized emotional sounds, the International Affective Digitized Sounds 2nd Edition (IADS-2) database [28], and found that the significant difference between pleasant and unpleasant sound responses was indicated by brain potentials from 200 milliseconds after the onset of the stimuli and the effect lasted beyond 1400 milliseconds after the stimuli [29]. It was also found that the frequency analysis in a 1.4 s window is insufficient for the classification of emotion. This suggests the importance of window size selection when measuring response to acoustic stimulus from EEG signals.

However, listening to a stream of music differs from attending a transient acoustic stimulus. A recent theory of musical emotions suggests the involvement of numerous mechanisms, including rhythmic entrainment, reward, aesthetic judgments, memory, and music anticipation, each of which emerges at a different time [30]. A previous study expanded the size of the window to 10 s to ensure the inclusion of mechanisms of anticipation as well as experience of the musical event and found the synchrony of the increment of frontal alpha asymmetry and a dominant change in musical features (such as motif, instruments, and pitch) within 5 s before to 5 s after the peak of the frontal asymmetry [31].

The experience of bodily changes leads to the affective feelings of pleasure and displeasure with some degree of arousal [32], which take some time to conceptualize and perceive automatically [33]. Then, it might require at least several seconds to perform emotional judgment and correctly annotate the perceived emotion [34]. The time-accuracy of annotation might also vary across subjects by cognitive availability.

Taking all the above into account, the latency of emotional response to sounds, diverse emerging time of different mechanisms involved in music listening, temporal resolution in emotion perception and annotation, and cognitive availability can be influential to indicating the appropriateness of the window size in EEG-based emotion recognition, which is not fully explored. Even though the size of the window was studied in EEG-based emotion recognition using feature-based learning [35,36], none of the studies have been focusing on CNN.

Apart from temporal information, spatial information is also crucial for the learning of CNN. In particular, CNN benefits from the locality of input and is successful in finding an edge in image classification tasks [37]. Therefore, the placement of adjacent electrodes in the input matrix for CNN can be impactful in learning, meaning that the accuracy in CNN-based learning from EEG signal might be improved by optimally re-arranging the order of EEG electrodes. Despite this promise, channel ordering received limited observation [38] and the results are often inconclusive [39]. For example, some studies have attempts of computing a connectivity index [40], using a dynamical graph CNN [41], or incorporating three-dimensional (3D) CNN [42,43].

Due to the lack of study of window size and channel ordering on CNN, we thus aim to investigate how changing window sizes and electrode order of EEG affect subject-independent emotion classification performance based on several CNN architectures in this study. In other words, the contribution of this research is being the first study that scrutinizes the effect of EEG spatiotemporal factors on the accuracy of emotion recognition performed by CNNs.

## 2. Background

### 2.1. Emotion Model

The two-dimensional (2D) emotion model was the model used to represent emotions in this study [3]. Emotions were formed from two values, the arousal and valence. Arousal represented the activation level of emotion, while valence indicated positivity or negativity. For example, sadness is a mixture of low arousal and negative valence. 

### 2.2. CNN

The CNN is one of the main categories of neural network that, literally, applies discrete convolution as its base concept [16]. In the network, there can be convolutional, pooling, dropout [44], and fully connected layers. These components together help the network learn and recognize patterns. The key part of CNN is the convolutional layer, where the convolution is computed. Mathematically, the layer uses discrete functions *f* and *g* to compute the output. Its formula is shown below:

(1) $$\left(f*g\right)\left[n\right]=\;\sum_{m=\;-\infty }^{\infty }f\left[m\right]g\left[n-m\right]$$

where *f* can be viewed as the layer’s kernel and *g* can be considered as the layer’s input. The layer performs element-wise multiplication of the kernel and the input, and then it adds all results up to obtain the output at *n*. The unique property of the CNN is that it can gain spatial knowledge, because, as can be seen from (1), when it calculates output at *n*, it takes data points around *n* (at any point *m*) into consideration. In other words, it incorporates neighboring knowledge when calculating the output at *n*. This ability has made it well-known in image classification. More information about CNN architectures and their implementation in this work can be found in Reference [45].

## 3. Materials and Methods

### 3.1. Data Collecting and Preprocessing

#### 3.1.1. Subjects

Experimental data were acquired from twelve students from Osaka University. All subjects were healthy men with a mean age of 25.59 years, standard deviation (SD, 1.69 years). Informed consent forms were obtained from all of the participants. Music tracks were used as stimulation and none of the participants ever had formal music education training. The music collection consisted of 40 MIDI files, comprising songs whose expected emotions were equally distributed over four quadrants in arousal-valence space. A majority of songs were drawn from previous studies on music-emotion [46,47]. Each song had only distinct instrument and tempo to circumvent influence from the lyrics. Subjects were directed to select 16 songs out of the collection. Hereby, the subjects were instructed to select eight songs with which they felt familiar and eight unfamiliar songs. To facilitate familiarity judging, our data collection software provided a function to play short (10 s) samples of songs to the subjects.

#### 3.1.2. Data Collecting Procedure

**Figure 1:**
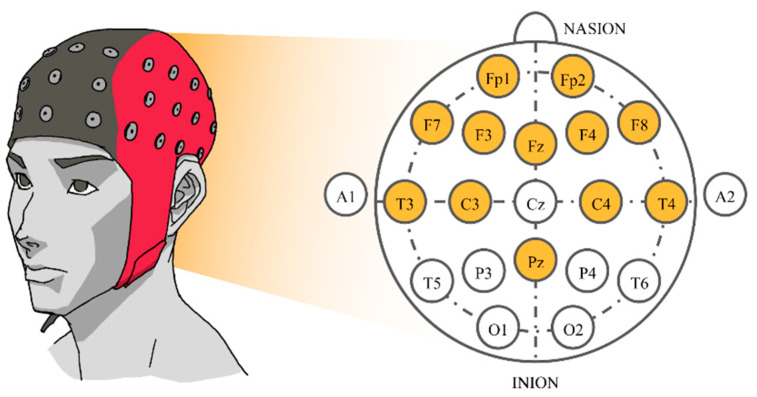
The 10–20 system of electrode placement showing the selected electrodes.

After selecting 16 songs, the subjects were instructed to listen to them. Songs were synthesized using Java Sound API’s MIDI package [48] and presented to the subjects via headphones. Each song was played for approximately 2 min. There was a 16 s silence interval between songs, reducing any carry-over effect between songs. While each subject was listening to the selected music track, their EEG signals were recorded concurrently. The sampling rate was set at 250 Hz. Signals were collected from twelve electrodes based on a Waveguard EEG cap [49]. The 10–20 international system with Cz as a reference electrode was employed. Those twelve electrodes were all located near the frontal lobe that plays an outstanding role in emotion regulation [50], i.e., Fp1, Fp2, F3, F4, F7, F8, Fz, C3, C4, T3, T4, and Pz (Figure 1).

Every electrode was adjusted to have an impedance of less than 20 kΩ. Sequentially, EEG signals were driven through a Polymate API532 amplifier and visualized by APMonitor, both developed by the TEAC Corporation [51]. A notch filter was set on the amplifier at 60 Hz to ensure that the power line artifact did not interfere with the analysis. Each subject was asked to close his eyes and stay still during the experiment to avoid generation of other unrelated artifacts. When a subject had finished listening to all 16 songs, the next step was to annotate their emotions. The EEG cap was detached, and the subjects had to listen to the same songs again in the same order. They labeled their feelings by continuously clicking on the corresponding spot in arousal-valence space displayed on a screen. The subjects were encouraged to continuously click at constant frequency (recommended to be every 2–3 s). The system recorded the arousal and valence data separately as the time-varying coordinates *x* and *y* in arousal-valence space, each of which ranges from −1 to 1.

#### 3.1.3. EEG Preprocessing

The frequency range analyzed in this work was 0.5–60 Hz. We employed a bandpass filter to cut off others which were not related. In addition, EEGLAB [52], an open-source MATLAB environment for EEG processing, was used to remove contaminated artifacts based on Independent Component Analysis (ICA) [53]. In particular, ICA was computed using the info-max algorithm [54], and the resultant independent components (ICs) were evaluated based on power spectral density (PSD), scalp topography, and location of the equivalent current dipole (fit by using Dipfit2.2 [55]). Specifically, we investigated the presence/absence of 1/f characteristic, spectral plateau above 25 Hz, and the peaks in alpha band (8–13 Hz) in PSD feature, the proximity of dipole if it falls inside or outside brain regions, and the direction of current reflected in scalp topography [56]. Then, we classified ICs into classes of brain, eye-movement, muscle, and other unrelated noises. Only brain-related ICs are remained and used to back-project to generate artifact-free EEG signals. When all antecedent steps were completed, signals were associated with emotion annotation via timestamps.

The mean and SD were calculated from the signal samples, and so we were able to perform feature scaling in terms of standardization (z-score). This helps the classification reduce error rates from high varying signal magnitudes [57].

**Figure 2:**
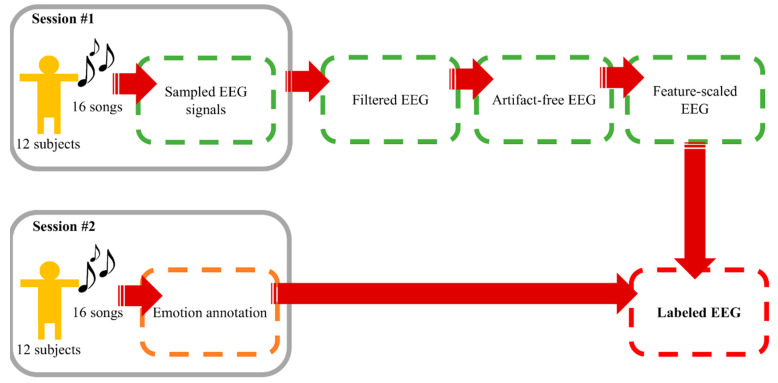
Data collecting and preprocessing procedure.

A number of current works on EEG-based emotion recognition aim to classify EEG signals into discrete classes of emotion owing to the fact that precise arousal-valence labelling requires immense understanding on arousal-valence space and a great extent of self-awareness of emotion [58]. Simplification by categorizing emotional labels into two or more classes is a common but effective practice. Following this motivation, arousal was divided into high and low, whereas valence was grouped into positive and negative. We considered emotion recognition as binary classification tasks of both components. Data from this process were considered the foundation materials for “Window Size Experiment” and “Electrode Sorting Experiment”. The process is summarized in Figure 2.

### 3.2. CNN Architectures

**Figure 3:**
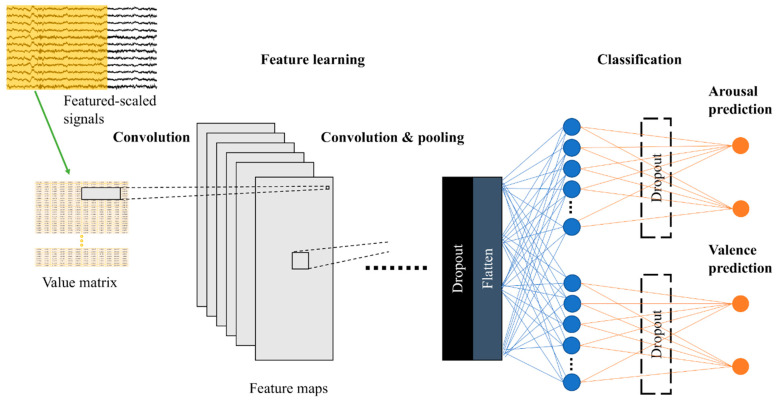
Model illustration.

The aim of this paper is to study CNN’s capabilities in general, so we reported results from various models (3Conv–6Conv) together with the average results. Their architectures are described in the table below (Table 1). Conv2D denotes two-dimensional (2D) convolutional layer, MaxPooling2D denotes 2D max pooling, and FC denotes fully connected layer. The input size is width × height, where width is the number of electrode channels (fixed at 12) and height equals to window size x sampling rate (fixed at 250 Hz). The outputs of these models are in the shape of 2 × 1 for predicting arousal (high or low) and valence (positive or negative), as shown in Figure 3. These networks had a little modification when testing electrode sorting. Please refer to the section “Electrode Sorting Experiment” and our GitHub repository [45] for more details on CNN architectures.

### 3.3. Window Size Experiment

Feature-scaled collected EEG signals from the previous part were sliced into timeframes in the range from 1 to 10 s, without overlapping time. Then, we trained and tested diverse CNNs on various window sizes according to networks described in Section 3.2. We obtained arousal and valence targets from the majority of user-annotated classes in each window. We applied leave-one-subject-out cross-validation (LOSO CV) to evaluate the experiment. During training, a set of instances was randomly selected within the training set to validate the training and accordingly avoid overfitting. Accuracy was defined as:

(2) $$Accuracy=\frac{TP+TN}{TP+FP+TN+FN}\times 100$$

where TP, TN, FP, and FN were denoted as the number of true positives, true negatives, false positives, and false negatives, respectively.

Self-reporting the emotion state can lead to an imbalance of the data. Therefore, we adopted the Matthews correlation coefficient (MCC) which takes class imbalance into account [59]. It can be calculated as:

(3) $$MCC=\frac{TP\times TN-FP\times FN}{\sqrt{\left(TP+FP\right)\left(TP+FN\right)\left(TN+FP\right)\left(TN+FN\right)}}$$

where all abbreviations are the same as in (2). The MCC ranges from −1 to 1, inferring all wrong to all correct predictions. This makes chance level prediction, which generally gains fairly good accuracy in the imbalance dataset, yield an MCC of zero.

### 3.4. Electrode Sorting Experiment

The performance could be affected by the channel arrangement, so we explored the three methodologies of the 3D physical order, maximal correlation-based order (MaxCBO), and minimal correlation-based order (MinCBO). The random order was employed as our benchmark. There were no changes from the architectures and feature-scaled signals mentioned earlier.

**Figure 4:**
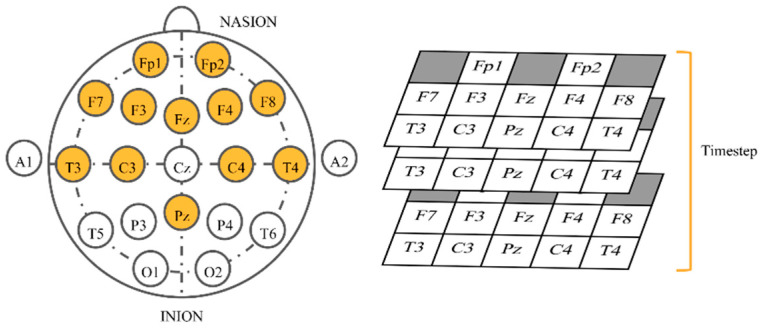
Input shape of three-dimensional (3D) physical order imitating the helmet.

For the 3D physical order, we applied a similar idea to that in References [42,60] to EEG-based emotion recognition by mimicking electrode placement on the real device. When gazing from the top view, electrodes were put in a 2D space. We stacked values placing them in this allocation in the order of time steps. Neighbor electrodes may have interrelated correlations that possibly help CNNs learn better. Classifiers were slightly modified to work with 3D inputs (Table 2). Conv3D denotes 3D convolutional layer, MaxPooling3D denotes 3D max pooling, and FC means fully connected layer. The input size (Figure 4) is width x height x depth, where width is fixed at 5, height is fixed at 3, and depth equals to window size x sampling rate (fixed at 250 Hz). The shape of outputs remained the same.

The MaxCBO was first proposed by Wen and colleagues to be used together with CNNs [38]. In fact, in their paper, two channel arrangement algorithms (maximizing adjacent information and maximizing overall information) were tested.

Both methods used the Pearson correlation coefficient (PCC) as their deciding factor, which can be calculated as:

(4) $$\rho_{xy}=\frac{\sum_{i=1}^{t}\left(x_{i}-\bar{x}\right)\left(y_{i}-\bar{y}\right)}{\sqrt{\sum_{i=1}^{t}{\left(x_{i}-\bar{x}\right)}^{2}}\sqrt{\sum_{i=1}^{t}{\left(y_{i}-\bar{y}\right)}^{2}}}$$

where $x_{i}$ represents the value of signal sampled from electrode x at time step i, and $\bar{x}$ is the mean value of all $x_{i}$. Similarly, $y_{i}$ and $\bar{y}$ possess the same definition for electrode y. From (4), *t* is the total samples for electrodes x and y. Thus, $\rho_{xy}$ denotes PCC between x and y. However, the algorithm considered $\left|\rho_{xy}\right|$, the absolute value of $\rho_{xy}$, to reflect the strength of the correlation. It was shown that the maximizing adjacent information algorithm outperformed another one [38], so we implemented it as a MaxCBO and presented those results in this work.

The MinCBO also used $\left|\rho_{xy}\right|$ as its deciding factor, but it would pick the least value first instead. We proposed this idea, which stemmed from the fact that some electrode pairs have very similar electric potentials that lead to high absolute PCC values. Placing channels with the highest possible $\left|\rho_{xy}\right|$ close together could be redundant. Wen and colleagues only maximized $\left|\rho_{xy}\right|$ [38], and, as far as we know, there has been no tests minimizing this coefficient, and so we decided to implement this idea.

The results from these three methods were evaluated and measured using the LOSO CV with a fixed window size of 4 s and 1 s of overlapping time. For our benchmark, we used the average results from 20 randomizations.

## 4. Results and Discussion

### 4.1. Window Size

By varying window sizes, we were able to see their effects on the classification performance (Table 3). Increasing the window frame could gain a higher performance in all CNN architectures. In addition, this trend applied to both the arousal and valence. However, this factor seemed to ameliorate the recognition of the valence more than the arousal, considering the valence had an MCC range of 0.1302 while the arousal had a range of 0.0951.

**Figure 5:**
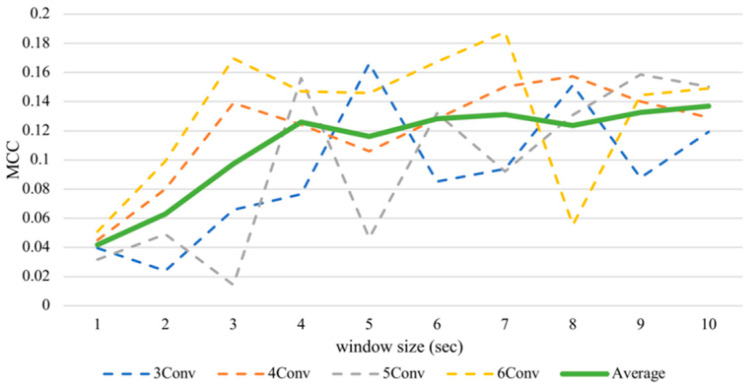
Subject-independent arousal MCC values with different window sizes.

Despite the fluctuation, the enlarging timeframe positively influenced arousal accuracy (Figure 5). From the average line, the performance increased significantly between one to four seconds. However, when the window sizes larger than four seconds were used, the slope of the graph became shallower. That means, on average, classifying arousal achieved the best outcome with a window size of ten seconds, obtaining 56.85% accuracy and an MCC value of 0.1369, while it executed the worst with a size of one second with an accuracy of 52.09% and an MCC value of 0.0418. There is a significant difference between these two MCC results proven by the Kruskal–Wallis test at *p* < 0.05, obtaining *H* = 4.3613.

**Figure 6:**
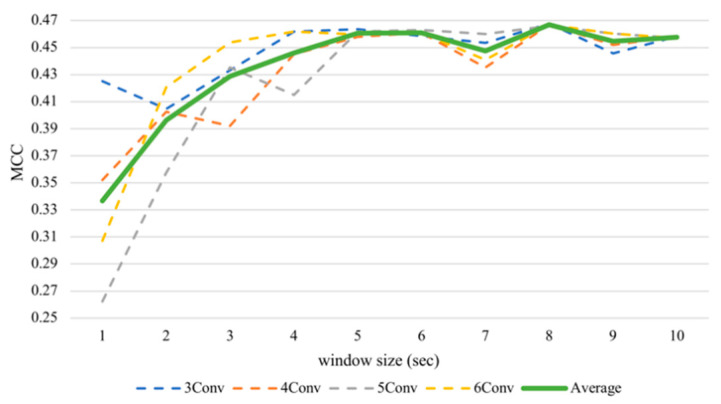
Subject-independent valence MCC values with different window sizes.

With respect to the valence, the average performance elevated sharply until five seconds (Figure 6) and then maintained this level until the end. The valence recognition had the best average performance with a window frame of eight seconds at 73.34% accuracy and an MCC value of 0.4669. It attained the lowest accuracy of 66.83% and an MCC of 0.3367 when using a one second window size. We analyzed the differences between results of the one second window and other sizes of the same architecture by performing the Wilcoxon signed-rank test. As there were multiple hypotheses tested (*m* = 9 comparisons per model × 4 models = 36), a multiple-testing correction was done using a false discovery rate (FDR) test as a correction procedure [61] where bolds in the table indicate significant differences after correction. It turned out that if we consider only one model per test (*N* = 12), arousal did not seem to make a significant difference, while valence made significant differences in some tests, especially after FDR correction. Table 3 has shown that, for valence classification, CNNs with more layers tend to gain significant improvements when increasing the window size, especially the 6Conv model. Results of 4Conv and 5Conv in general did not demonstrate significant differences from one second before FDR correction, except the results from eight seconds, which outperformed other window sizes and could show statistical differences. 

According to our results, there could be immense variations between signals from each subject. Expanding the window might help decrease differences among them. In other words, gazing in a larger period could reduce fluctuation among instances from distinct subjects; hence, a wider window size may give a better performance when employing the LOSO CV. In particular, extending the timeframe could markedly help during the first one to four seconds for arousal recognition, and one to five seconds for valence recognition, on average. This also suggested that window size effect could overshadow the model complexity because the trend could be found in every classifier. However, model complexity did help the performance regarding valence recognition and the statistical differences. Models with high complexity tend to have significant valence recognition improvement compared to results based on the one second window. This is intuitive, because models with more layers have more parameters to train and, thus, can handle more complex features in EEG.

Moreover, if we compare results to our previous work [12], which examined the traditional support vector machine (SVM) with linear, polynomial, and RBF kernel based on the same EEG dataset, window size was less influential on the SVM than on the CNN (Supplementary File). The arousal had an accuracy range of only 2.11% and the valence obtained a range of 2.52% in the subject-independent task. 

**Figure 7:**
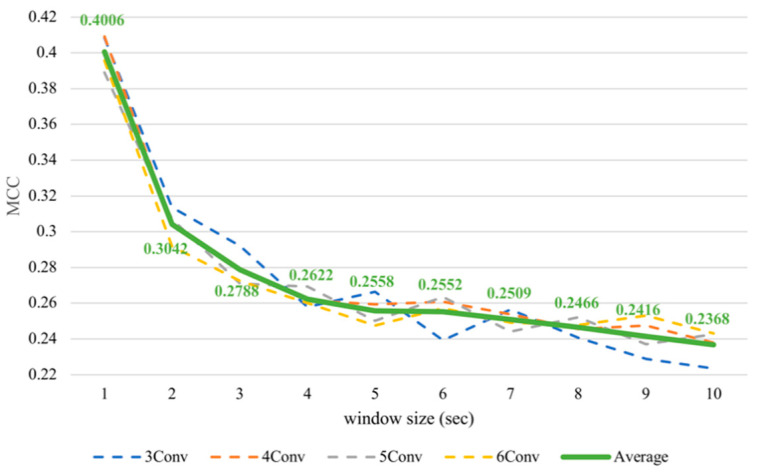
Subject-dependent arousal MCC values with different window sizes.

**Figure 8:**
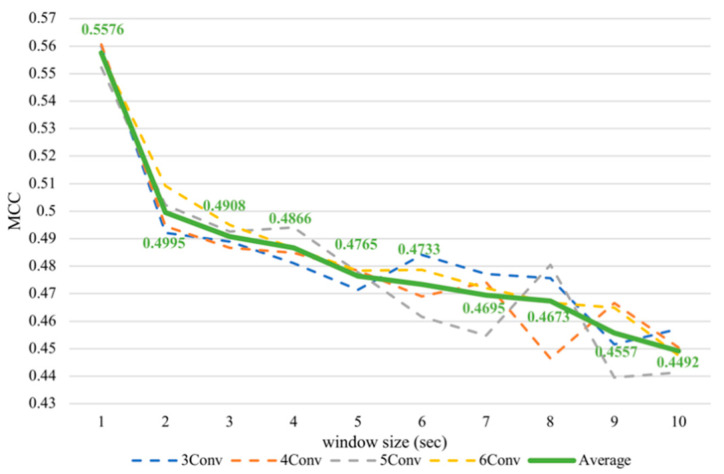
Subject-dependent valence MCC values with different window sizes.

Nevertheless, it was noticeable that the subject-dependent task from the previous work [12] had a completely converse trend from cross-subject results in this paper. Therefore, we conducted subject-dependent experiments on classifying arousal and valence based on CNNs. Each experiment was validated using 10-fold cross-validation. The results showed that training on CNNs obtained a similar trend as training on SVM, as can be seen in Figure 7 and Figure 8. In fact, testing in a subject-independent fashion can produce a test set, which has a greater similarity to the training set considering that each subject was tested separately. Thus, shrinking the window size would make them more closely resemble the test set and create more training instances, too. Hence, this could make the subject-dependent problem have a different outcome.

There may be concerns in the unequal amount of the training and test instances for each window size. While keeping the usage of the entire dataset, varying the length of windows produced a biased number of instances. Although under-sampling should be performed to avoid evaluation unfairness, results have shown that experiments with less training and test instances (those with larger window size) nevertheless gained higher accuracy. Future work should therefore include follow-up work designed to collect more data balancing both classes or should incorporate existing publicly available datasets whose experiments are reminiscent of our work that allows continuous annotation of emotion.

To demonstrate the attractiveness of our proposed method that considers the size of the window, we conducted an experiment to compare our results with recent works in EEG-based emotion recognition. Despite the difference in annotation continuity in the experiment, we selected several recent works that proposed algorithms to improve subject-independent classification accuracy of SEED [62] and DEAP [63] datasets. As direct comparison with the results in those publications cannot be made due to the difference in window size and annotation, we implemented such algorithms and applied them to our dataset with the fixation of window size of 8 s, which achieved good performance in both arousal and valence classification, as shown above.

First, we implemented the Dynamic Sampling Entropy method [64] that achieved high accuracy with the SEED dataset. Following the original paper, we used a sub-sliding window of 4 s length with 50% overlap for our 8 s segment data, and we used sample entropy [65] for feature extraction and SVM with RBF kernel function for classification. Second, we followed a recent successful work in the DEAP dataset by implementing deep neural network for classifying features of PSD peak of intrinsic mode functions (IMF) of EEG data and the first derivative of IMF [66]. We used the same architecture that achieved best accuracy in the paper, with Relu as the activation function and gradient descent as an optimization method (IMF-DNN). We further improved the accuracy by varying activation function and optimization methods and reported the best accuracy as the improved version of this method (IMF-DNN-improved). Third, we applied transfer learning to our dataset which has high potential to mitigate inter-subject variability between subjects in EEG data. Following a recent work that explored the best transfer learning algorithms for both SEED and DEAP [67], we implemented maximum independence domain adaptation (MIDA) [68] and Transfer Component Analysis (TCA) [69], where we used linear kernel, mu = 1, and we searched the best latent subspace dimension from {5, 10, …, 30}, identical to the hyperparameters in the original paper. Nevertheless, we applied SVM with linear kernel with PSD features as the method and features also achieved comparable results, as demonstrated in the original publication of SEED [62]. For our method, we presented the classification and MCC of the best performing architecture when the window size is 8 s (Table 3).

Table 4 shows the comparison between the existing methods and our proposed method. The results suggest that our method outperformed the state-of-the-art approach in valance classification. Although the transfer learning (TCA) achieved superior accuracy to our method, the higher MCC of our method indicates that it successfully learned to classify patterns with less susceptibility to one-class random guessing. Besides, our method also involves minimal feature engineering. It is also worthy to note that the main goal of our research is not to propose the best classification algorithm to recognize emotional classes but to study the effect of parameters including window size and channel arrangement. Still, the results of our method can outperform the existing methods when fixing the window size to the length that achieved decent performance in our experiment. This evidence emphasizes the importance of investigating the optimal window size when implementing CNN-based emotion recognition using EEG signal. Yet, future studies are encouraged to involve more sophisticated algorithms in deep learning to further improve the classification accuracy and MCC.

### 4.2. Electrode Sorting

Electrode sorting appeared to have a less marked effect on the classification than the window size (Table 5). All sorting methods that we tested gained modest differences from that with a random order in both the arousal and valence recognition. However, nearly all of our algorithms could outperform the results from the random order, on average. We investigated the statistical differences between the random order and other sorting algorithms of the same model based on the Wilcoxon signed-rank test (*N* = 12). Again, an FDR correction was done to correct the resultant *p*-values as there were 12 hypotheses (3 comparisons per model × 4 models) involved where bolds in the table indicate significant differences after correction. The results showed that the arousal recognition had relatively arbitrary outcomes. That is, we could not observe any trend in terms of statistical improvement of the results. Nevertheless, for valence classification, we can see that mostly 3D physical order had significant differences compared to the random order. The MinCBO also had significant improvement overall. The MaxCBO, on the other hand, did not have significant differences from random.

As reported in the arousal results, the MinCBO had the best performance, with an average accuracy of 56.92% and MCC value of 0.1384. The MaxCBO was the worst among all the tested systems, obtaining an accuracy of 56.12% and an MCC value of 0.1223, but they could still perform better than the outcomes from the random order. Even though all the mean results of the three orders outperformed the random order, some outputs from some settings acquired less precision than those from the random algorithm.

The valence classification task acquired the greatest performance using the 3D physical order, at 72.94% accuracy and an MCC value of 0.4588. The MaxCBO, once again, had the worst results, with an accuracy of 71.70% and an MCC value of 0.4340, which were even lower than the randomizing. The MinCBO achieved slightly better outputs than the random order.

In spite of having no significant differences from our benchmark, the MaxCBO always achieved the lowest performance among the other three algorithms in both the arousal and valence. This might be caused by the signal resemblance of the juxtaposed electrodes. It is possible that CNNs could not gain a competitive amount of information when placing similar signals closely. Although the networks had multiple layers, latter layers were designed to catch higher-level features and so they might not be able to capture meticulous features. Putting electrodes with a stark contrast near each other could potentially help CNNs learn detailed features more efficiently. This might be the reason why the 3D physical MinCBOs had a higher prediction accuracy.

**Figure 9:**
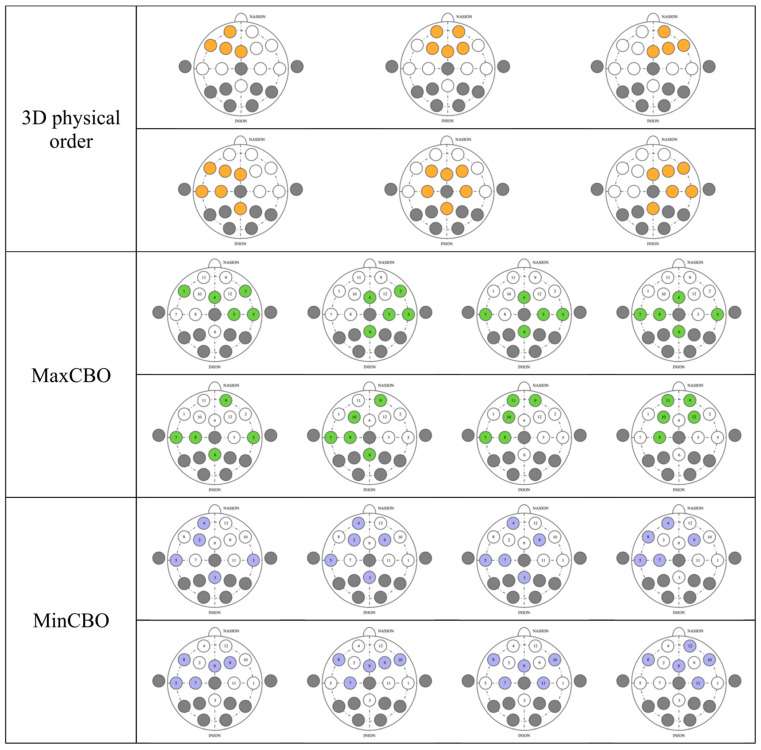
Channel selection before feeding into first-layer kernels in various electrode sorting algorithms.

The asymmetry of brain activities between the left and right hemispheres over the frontal cortex potentially implicates the study’s outcome [63]. Even though the theory is still debatable, brain activity imbalance tends to have a strong association with valence, while arousal seems to be less related [70]. Our experiments, incorporating CNNs, potentially calculated asymmetry indexes, and this could be the reason why results from valence recognition were much more superior to those from the arousal recognition, which only gained an accuracy slightly above 50%. Since the electrode order could be crucial in capturing meticulous low-level features, from our investigation, every arrangement was capable of catching differences in the hemisphere activity in accordance with the theory. We visualized electrode selection in the first CNN layers for a better understanding (Figure 9). Every type had several kernels that comprised channels from both the left and right hemispheres, letting the models calculate cross-hemisphere features elaborately. Even the random order also had a high propensity to place electrodes from both sides near each other, because the first-layer kernels from our models picked every five contiguous channels from the total of twelve channels. This gave a very high chance for the models to select electrodes from both sides and calculate the differences between them. Thus, CNNs can calculate asymmetry indexes in every arrangement, and this eventually makes their performance in the valence clearly better than in the arousal, including the random order. In our previous SVM experiment [12], valence was found to outperform arousal as well, since asymmetry indexes were included by manual feature extraction. However, CNNs in this study still had a higher valence classification accuracy than SVM. Future study is awaited to investigate the underlying mechanism of cross-hemispheric asymmetry, where studies on functional connectivity and effective connectivity [71] may elucidate the neural underpinnings of the phenomenon and help guide selecting the right pairs of electrodes when establishing CNN architecture.

### 4.3. Future Research Direction

Window size was found to clearly influence the emotion recognition using CNNs. The process of emotion conceptualization [33] and delivering the right emotion annotation [34] might require several seconds, as evident in our results that demonstrate a huge improvement until expanding the window size beyond 3 s. The mechanism on how this process unfolds over the course of time is worth to be carefully studied in future work.

Even though, in contrast, the electrode sorting algorithm did not exhibit a clear impact in this study, it might be that all of our models could benefit from asymmetry brain activity indexes. Hence, we should find a way to establish that our models actually learn features relating to brain activity imbalance, perhaps by feature reconstruction, so we could design electrode sorting algorithms, together with an appropriate CNN architecture, that could increase the valence accuracy.

However, arousal recognition is still a difficult task. The difference in performance might be due to the distinct neurobiological processes of arousal and valance. It is evident that both the window size and electrode arrangement showed only a trivial effect in enhancing inter-subject correctness. Arousal might correspond to the frequency band in the central scalp [72], but this study is still highly controversial. Despite a lack of rigorous per-subject investigation, Chen and colleagues also found that CNN could learn frequency-related features, as proven from the reconstruction technique based on the subject who gained the best arousal performance [18]. Modeling the CNN architecture, window size calculation, and electrode sorting in order to specifically extract band-power-related features would be an interesting research direction for future work in arousal. Extensive exploration of CNN architectures can also be of interest. For instance, a much deeper CNN might successfully learn to sort electrodes in a way that yields comparable architecture as the MaxCBO or MinCBO approach by capturing spatial correlation in the added layers, despite the compromise with more parameters. Likewise, a memory-based approach, for example, recurrent neural network and long short-term memory, might accomplish learning a temporal relationship but with an increased number of hyperparameters.

The implication of the present study may not be limited to EEG-based emotion recognition using musical stimuli. The effect of window size and electrode sorting might apply to other circumstances where different varying stimuli are involved, such as a stream of images, videos, and narratives. A variety of emotion-triggering sources might induce different brain responses at different timeframes. Future work should therefore investigate the window size and electrode sorting when implementing CNN in other experimental paradigms in order to validate our hypothesis.

Regarding the practicality, future research should also aim to enable the application of real-time EEG-based emotion recognition in real-life scenarios. In particular, the present study employed the info-max algorithm to perform ICA decomposition and resultant ICs were manually evaluated based on IC’s properties. The long processing time of ICA decomposition and offline laborious process in IC assessment hinders the automation of the system toward online detection of emotion. Recently, the optimized online recursive ICA algorithm (ORICA) [73] was developed, offering the automatic IC evaluation and non-brain source removal, instead of reliance on the traditional ICA approach. Alternatively, artifact subspace reconstruction (ASR) [74,75] can be incorporated to clean EEG signals in real-time, albeit with the necessity to pre-record baseline period. Future studies are awaited to validate the applicability of these approaches to our work with the aim to migrate from offline to online emotion recognition based on EEG.

## 5. Conclusions

In this paper, the window size selection and electrode sorting effects on the CNN classification performance in the EEG-emotion recognition task were evaluated by collecting EEG signals from twelve subjects. Evaluation of the LOSO CV was performed from the preprocessed signals in a subject-independent fashion, focusing on model generalization to unseen subjects. We used the average results from various CNN architectures so that the results were the representatives of CNNs in general. Widening window size was shown to improve the average accuracy, especially for arousal in the first four seconds and valence in the first five seconds. Increasing the window size could possibly decrease variability across subjects, and, therefore, give the emotion classification a better outcome. With respect to the electrode arrangement, the sorting algorithms tested in this study appeared to only have a trivial effect on the performance. Mainly, our limitation is that we could not identify exactly what the model learned. However, we suspect that all classifiers we assessed might have captured asymmetry indexes, which could potentially assist the valence accuracy, but not the arousal accuracy. In consequence, we should try to establish if inter-hemisphere features were actually learned for valence recognition. Furthermore, we should address band power features, since they may be related to arousal recognition. With this knowledge, researchers have a better possibility to design an improved CNN, a suitable window size selection, and electrode order in order to improve the performance of this task in the future.

## Figures and Tables

**Table 1:** Network architectures.

3Conv		4Conv		5Conv		6Conv	
Conv2D (5 × 5) × 32		Conv2D (5 × 5) × 32		Conv2D (5 × 5) × 32		Conv2D (5 × 5) × 32	
Conv2D (3 × 3) × 32		Conv2D (3 × 3) × 32		Conv2D (2 × 2) × 32		Conv2D (2 × 2) × 32	
MaxPooling2D 2 × 2		MaxPooling2D 2 × 2		Conv2D (2 × 2) × 32		Conv2D (2 × 2) × 32	
Conv2D (3 × 3) × 64		Conv2D (2 × 2) × 64		MaxPooling2D 2 × 2		MaxPooling2D 2 × 2	
Dropout 0.5		Conv2D (2 × 2) × 64		Conv2D (2 × 2) × 64		Conv2D (2 × 2) × 64	
FC 128 × 1	FC 128 × 1	Dropout 0.5		Conv2D (2 × 2) × 64		Conv2D (2 × 2) × 64	
Dropout 0.5	Dropout 0.5	FC 128 × 1	FC 128 × 1	Dropout 0.5		Conv2D (2 × 1) × 64	
FC 2 × 1	FC 2 × 1	Dropout 0.5	Dropout 0.5	FC 128 × 1	FC 128 × 1	Dropout 0.5	
		FC 2 × 1	FC 2 × 1	Dropout 0.5	Dropout 0.5	FC 128 × 1	FC 128 × 1
				FC 2 × 1	FC 2 × 1	Dropout 0.5	Dropout 0.5
						FC 2 × 1	FC 2 × 1

**Table 2:** Network architectures with 3D inputs.

3Conv		4Conv		5Conv		6Conv	
Conv3D (9 × 2 × 3) × 32		Conv3D (9 × 2 × 3) × 32		Conv3D (9 × 2 × 3) × 32		Conv3D (9 × 2 × 3) × 32	
Conv3D (3 × 2 × 3) × 32		Conv3D (3 × 2 × 3) × 32		Conv3D (3 × 2 × 3) × 32		Conv3D (3 × 2 × 3) × 32	
MaxPooling3D 4 × 1 × 1		MaxPooling3D 4 × 1 × 1		Conv3D (3 × 1 × 1) × 64		Conv3D (3 × 1 × 1) × 64	
Conv3D (3 × 1 × 1) × 64		Conv3D (3 × 1 × 1) × 64		MaxPooling3D 4 × 1 × 1		MaxPooling3D 4 × 1 × 1	
Dropout 0.5		Conv3D (3 × 1 × 1) × 64		Conv3D (3 × 1 × 1) × 64		Conv3D (3 × 1 × 1) × 64	
FC 128 × 1	FC 128 × 1	Dropout 0.5		Conv3D (3 × 1 × 1) × 64		Conv3D (3 × 1 × 1) × 64	
Dropout 0.5	Dropout 0.5	FC 128 × 1	FC 128 × 1	Dropout 0.5		Conv3D (3 × 1 × 1) × 64	
FC 2 × 1	FC 2 × 1	Dropout 0.5	Dropout 0.5	FC 128 × 1	FC 128 × 1	Dropout 0.5	
		FC 2 × 1	FC 2 × 1	Dropout 0.5	Dropout 0.5	FC 128 × 1	FC 128 × 1
				FC 2 × 1	FC 2 × 1	Dropout 0.5	Dropout 0.5
						FC 2 × 1	FC 2 × 1

**Table 3:** Classification accuracy (top row) and MCC (in brackets) based on varying window size. Note that * denotes significant difference at *p* < 0.05 (uncorrected), ** denotes that at *p* < 0.01 (uncorrected) when comparing to training on window size of 1 s, and bold indicates significant difference at FDR-corrected *p*-value (*q*-value) < 0.05.

		Window Size (s)									
		1	2	3	4	5	6	7	8	9	10
Arousal	3Conv	51.97 (0.0395)	51.20 (0.0240)	53.30 (0.0660)	53.83 (0.0766)	58.31 (0.1661)	54.26 (0.0853)	54.70 (0.0940)	57.57 (0.1514)	54.39 (0.0878)	55.96 (0.1192)
	4Conv	52.25 (0.0451)	54.01 (0.0801)	56.95 (0.1391)	56.22 (0.1243)	55.30 (0.1060)	56.41 (0.1282)	57.52 (0.1504)	57.87 (0.1573)	57.00 (0.1401)	56.46 (0.1291)
	5Conv	51.59 (0.0317)	52.45 (0.0491)	50.71 (0.0143)	57.81 (0.1561)	52.32 (0.0463)	56.60 (0.1321)	54.61 (0.0922)	56.54 (0.1308)	57.93 (0.1586)	57.52 (0.1504)
	6Conv	52.55 (0.0509)	54.95 (0.0991)	58.48 (0.1695)	57.35 (0.1470)	57.29 (0.1458)	58.36 (0.1673)	59.38 (0.1877)	52.77 (0.0553)	57.22 (0.1444)	57.45 (0.1489)
	Average	52.09 (0.0418)	53.15 (0.0631)	54.86 (0.0972)	56.30 (0.1260)	55.80 (0.1161)	56.41 (0.1282)	56.55 (0.1311)	56.19 (0.1237)	56.64 (0.1327)	56.85 (0.1369)
Valence	3Conv	71.26 (0.4251)	**70.23 (0.4046)**	71.66 (0.4333)	73.09 (0.4617)	73.18 (0.4635)	72.93 (0.4586)	72.67 (0.4534)	**73.39 (0.4678)**	72.29 (0.4457)	72.91 (0.4582)
	4Conv	67.60 (0.3521)	70.12 (0.4025)	69.60 (0.3921)	72.25 (0.4450)	**72.89 (0.4579)**	**73.05 (0.4609)**	71.76 (0.4352)	73.34 (0.4669) *	**72.60 (0.4520)**	**72.91 (0.4582)**
	5Conv	63.12 (0.2623)	67.87 (0.3575)	**71.76 (0.4352)**	70.75 (0.4149)	**73.09 (0.4619)**	**73.15 (0.4630)**	**72.10 (0.4599)**	73.30 (0.4660) *	**73.02 (0.4604)**	**72.86 (0.4571)**
	6Conv	65.36 (0.3072)	71.03 (0.4206)	72.68 (0.4537) *	73.08 (0.4617) **	**72.98 (0.4596)**	73.04 (0.4609) *	72.05 (0.4409) *	73.34 (0.4669) **	73.02 (0.4604) *	72.85 (0.4570) *
	Average	66.83 (0.3367)	69.82 (0.3963)	71.43 (0.4286)	72.29 (0.4458)	73.04 (0.4607)	73.04 (0.4608)	72.37 (0.4473)	73.34 (0.4669)	72.73 (0.4546)	72.88 (0.4577)

**Table 4:** The comparison of emotion classification results between the existing methods and our method showing the accuracy (%) and MCC (in brackets).

Methods	Arousal	Valence
Dynamic Sampling Entropy	50.10 (−0.0427)	67.15 (0.0940)
IMF-DNN	53.71 (−0.0306)	71.39 (−0.0061)
IMF-DNN-improved	55.30 (0.0148)	71.47 (0.0117)
MIDA	56.97 (0.0059)	63.42 (0.0152)
TCA	61.29 (0.0270)	62.68 (0.0270)
Our Method	57.87 (0.1573)	73.39 (0.4678)

**Table 5:** Accuracy (top row) and MCC (in brackets) based on multiple sorting algorithms. Note that * denotes significant difference at *p* < 0.05 (uncorrected), ** denotes that at *p* < 0.01 (uncorrected), and bold indicates significant difference at FDR-corrected *p*-value (*q*-value) < 0.01.

		Architecture				
		3Conv	4Conv	5Conv	6Conv	Average
Arousal	Random order	54.50 (0.0900)	55.71 (0.1142)	56.01 (0.1202)	55.93 (0.1187)	55.54 (0.1108)
	3D physical order	56.41 (0.1282)	56.44 (0.1288)	53.79 (0.0758)	59.41 (0.1882) **	56.51 (0.1303)
	MaxCBO	56.58 (0.1315)	56.16 (0.1231)	55.81 (0.1163)	55.92 (0.1183)	56.12 (0.1223)
	MinCBO	56.60 (0.1321) *	55.95 (0.1191)	57.46 (0.1491)	57.67 (0.1534)	56.92 (0.1384)
Valence	Random order	71.88 (0.4377)	72.08 (0.4416)	72.65 (0.4530)	72.95 (0.4590)	72.39 (0.4478)
	3D physical order	72.56 (0.4511) *	73.06 (0.4612) *	73.08 (0.4615) **	73.06 (0.4612)	72.94 (0.4588)
	MaxCBO	71.42 (0.4283)	71.02 (0.4204)	71.31 (0.4262)	73.06 (0.4612)	71.70 (0.4340)
	MinCBO	73.04 (0.4609) **	72.88 (0.4575) *	72.01 (0.4402)	73.06 (0.4612)	72.75 (0.4550)

## Data Availability

The data presented in this study are available on reasonable request from the corresponding author. The data are not publicly available due to confidentiality and data-archiving agreements.

## Supplementary Materials

The implementation of the CNNs in this study can be found at https://github.com/Gpanayu/EmoRecogKeras. Additional results are available online at https://www.mdpi.com/1424-8220/21/5/1678/s1 Table S1: averaged subject-independent emotion classification accuracies across subjects, Figure S1: averaged subject-independent emotion classification accuracies across subjects.
