# Supplementary material for: A Comparative Study of Window Size and Channel Arrangement on EEG-Emotion Recognition Using Deep CNN
Source: Sensors (Basel). 2021 Mar 1;21(5):1678. doi: 10.3390/s21051678 (PMC7957771; doi:10.3390/s21051678)
Supplement: Supplementary file 1 [file sensors-21-01678-s001.pdf]

Table A1: averaged subject-independent emotion classification accuracies across subjects

Classification	Method	Window size (s)								
		**2**	**3**	**4**	**5**	**6**	**7**	**8**	**9**	**10**
Arousal	SVM-Linear	47.04	46.80	46.73	46.90	46.14	46.93	46.96	47.40	48.25
	SVM-Polynomial	48.23	49.02	48.79	48.35	49.50	48.91	48.39	49.37	49.88
	SVM-RBF*	44.09	44.05	43.70	43.96	45.17	44.68	44.64	44.72	44.88
	This study	**53.15**	**54.86**	**56.30**	**55.80**	**56.41**	**56.55**	**56.19**	**56.64**	**56.85**
Valence	SVM-Linear	51.69	50.53	51.52	50.23	50.12	50.80	50.06	50.24	49.20
	SVM-Polynomial	51.18	50.73	51.33	51.23	49.62	50.72	50.39	49.75	49.24
	SVM-RBF*	52.95	53.89	54.87	54.84	53.66	54.90	55.47	54.88	54.46
	This study	**69.82**	**71.43**	**72.29**	**73.04**	**73.04**	**72.37**	**73.34**	**72.73**	**72.88**

* The results of SVM with RBF kernel are also presented in https://arxiv.org/abs/1611.10120

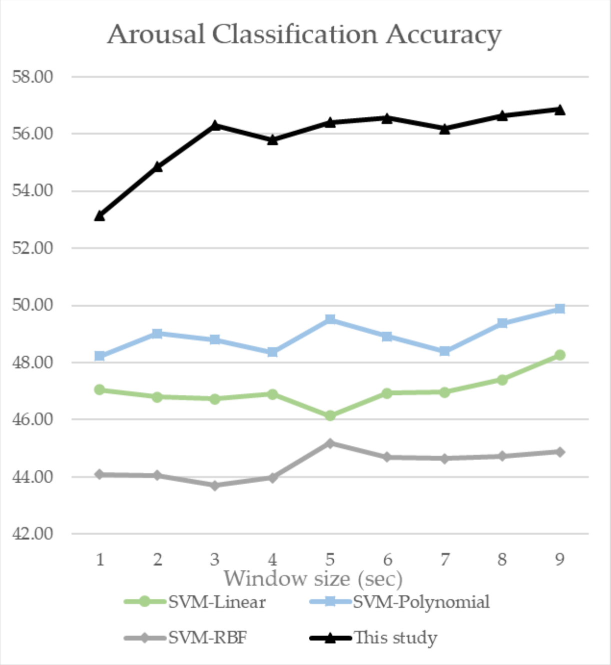

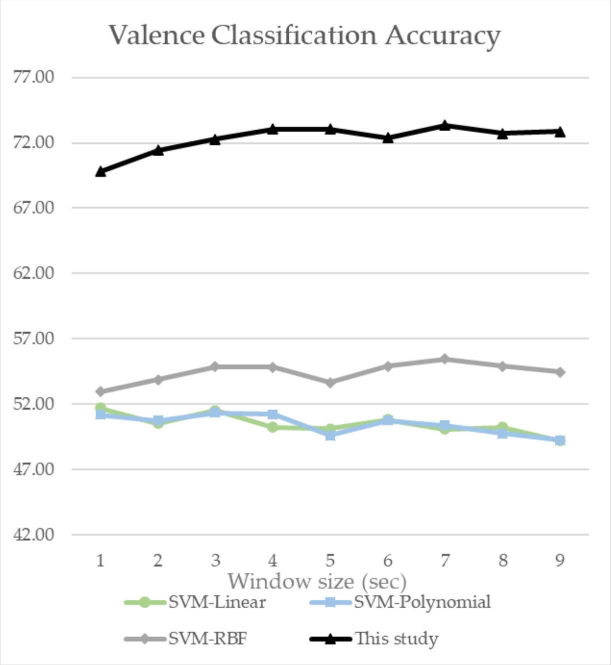

Figure A1: averaged subject-independent emotion classification accuracies across subjects
